# Evolution of and Horizontal Gene Transfer in the *Endornavirus* Genus

**DOI:** 10.1371/journal.pone.0064270

**Published:** 2013-05-07

**Authors:** Dami Song, Won Kyong Cho, Sang-Ho Park, Yeonhwa Jo, Kook-Hyung Kim

**Affiliations:** Department of Agricultural Biotechnology, Plant Genomics and Breeding Institute, Institute for Agriculture and Life Sciences, College of Agriculture and Life Sciences, Seoul National University, Seoul, Republic of Korea; Soonchunhyang University, Republic of Korea

## Abstract

The transfer of genetic information between unrelated species is referred to as horizontal gene transfer. Previous studies have demonstrated that both retroviral and non-retroviral sequences have been integrated into eukaryotic genomes. Recently, we identified many non-retroviral sequences in plant genomes. In this study, we investigated the evolutionary origin and gene transfer of domains present in endornaviruses which are double-stranded RNA viruses. Using the available sequences for endornaviruses, we found that *Bell pepper endornavirus*-like sequences homologous to the glycosyltransferase 28 domain are present in plants, fungi, and bacteria. The phylogenetic analysis revealed the glycosyltransferase 28 domain of *Bell pepper endornavirus* may have originated from bacteria. In addition, two domains of *Oryza sativa endornavirus*, a glycosyltransferase sugar-binding domain and a capsular polysaccharide synthesis protein, also exhibited high similarity to those of bacteria. We found evidence that at least four independent horizontal gene transfer events for the glycosyltransferase 28 domain have occurred among plants, fungi, and bacteria. The glycosyltransferase sugar-binding domains of two proteobacteria may have been horizontally transferred to the genome of *Thalassiosira pseudonana*. Our study is the first to show that three glycome-related viral genes in the genus *Endornavirus* have been acquired from marine bacteria by horizontal gene transfer.

## Introduction

Eukaryotic genomes have acquired genetic information through two different mechanisms throughout the course of evolution. The first mechanism is vertical gene transfer, in which the progeny receives genetic information from their ancestors, such as their parents. The second mechanism is horizontal gene transfer (HGT), which is the transfer of genetic information between unrelated species [1]. Evidence for HGT events has frequently been observed in prokaryotes and eukaryotes [2]–[5]. Numerous studies have suggested that HGT is one of important keys to understanding the evolution of prokaryotic and eukaryotic genomes [1], [3].

One frequent HGT event might be between a virus and the host [6]. Among the many known viruses, the retroviruses can easily integrate their viral genes or genomes into the host chromosomes because these RNA viruses utilize a reverse transcriptase to produce DNA from the RNA genome for their replication in a host cell [7], [8]. Consequently, a large number of retroviral sequences are found in eukaryotic genomes through sequencing and comparative analyses [9]. Endogenous hepadnaviruses have been discovered in the genomes of passerine birds, which include more than half of all bird species [10]. Previous studies have also identified endogenous pararetroviruses (EPRVs) in plant genomes [11]–[13]. EPRVs integrate into plants' nuclear genomes and become part of the plants genomes as the result of evolutionary forces [14].

Recently, several studies have demonstrated that non-retroviral sequences can also be integrated into eukaryotic genomes [15]–[17]. For instance, non-retroviral elements homologous to sequences in *Bornavirus*, *Filovirus*, *Circovirus*, and *Parvovirus* have been discovered in the genomes of several mammalian species [15]. The integration of non-retroviral RNA virus sequences (NRVSs) has also been demonstrated for several plant genomes, and multiple integration events for non-retroviral sequences into different plant lineages have been identified [16].

The members of the genus *Endornavirus* are not retroviruses, and this genus was recently created as a new genus of double-stranded (ds) RNA viruses in the family *Endornaviridae* by the International Committee on Taxonomy of Viruses (ICTV) [18]. The genomes of endornaviruses are linear dsRNAs of 9.8–17.6 kb in length and have only one open reading frame (ORF) [19]. These ORFs normally encode a single polypeptide that is thought to be processed by a proteinase, and the genome contains conserved motifs, including an RNA-dependent RNA polymerase (RdRp) and viral RNA helicases (Hel) [20]. Endornaviruses seem not to form true virions and are usually present at a low copy number [21]. These viruses have been found in plants, fungi, and protists [22].

Recently, our group identified several viral sequences that are homologous to plant genes. The gene transfer of such endogenous viral sequences might have occurred from the virus to the host or from the host to the virus. In this study, we obtained strong evidence for gene transfer between the virus and the host using the endornaviruses as a model. Based on these results, we propose a hypothesis related to the evolutionary origins and horizontal gene transfer of endornaviral genes.

## Materials and Methods

### Identification of endornavirus-like sequences in plant proteomes

We retrieved all 52 nucleotide sequences for 13 endornavirus species in the GenBank database of the National Center for Biotechnology Information (NCBI) (http://www.ncbi.nlm.nih.gov/). The full genome sequences of eight endornaviruses, *Bell pepper endornavirus* (NC_015781), *Helicobasidium mompa endornavirus 1* (NC_013447.1), *Oryza sativa endornavirus* (NC_007647.1), *Oryza rufipogon endornavirus* (NC_007649.1), *Vicia faba endornavirus* (NC_007648.1), *Phytophthora endornavirus 1* (NC_007069.1), *Tuber aestivum* endornavirus (NC_014904.1), and *Gremmeniella abietina* type B RNA virus XL1 (NC_007920.1), as well as the full amino acid sequences of *Chalara elegans* endornavirus 1 (ADN43901), were retrieved from NCBI. In parallel, we retrieved the whole proteome sequences for 30 plant species from Phytozome v. 7.0. (http://www.phytozome.net). The stand-alone BLAST program (Ver. 2.2.25) was downloaded from NCBI and installed on a computer running Windows 7 (64-bit). To find endornavirus-like sequences, we performed BLASTX and BLASTP searches with 1e-5 as the E-value cutoff against databases for plant proteomes, expressed sequence tags (ESTs), transcriptome shotgun assembly (TSA), and bacterial genomes. To detect predicted conserved domains in each endornavirus, the full-length amino acid sequences were subjected to analysis with the SMART program (http://smart.embl-heidelberg.de/), and sequence data associated with known domains were retrieved from the Pfam database (http://pfam.sanger.ac.uk/).

### Sequence alignment and phylogenetic analysis

**Figure 1:**
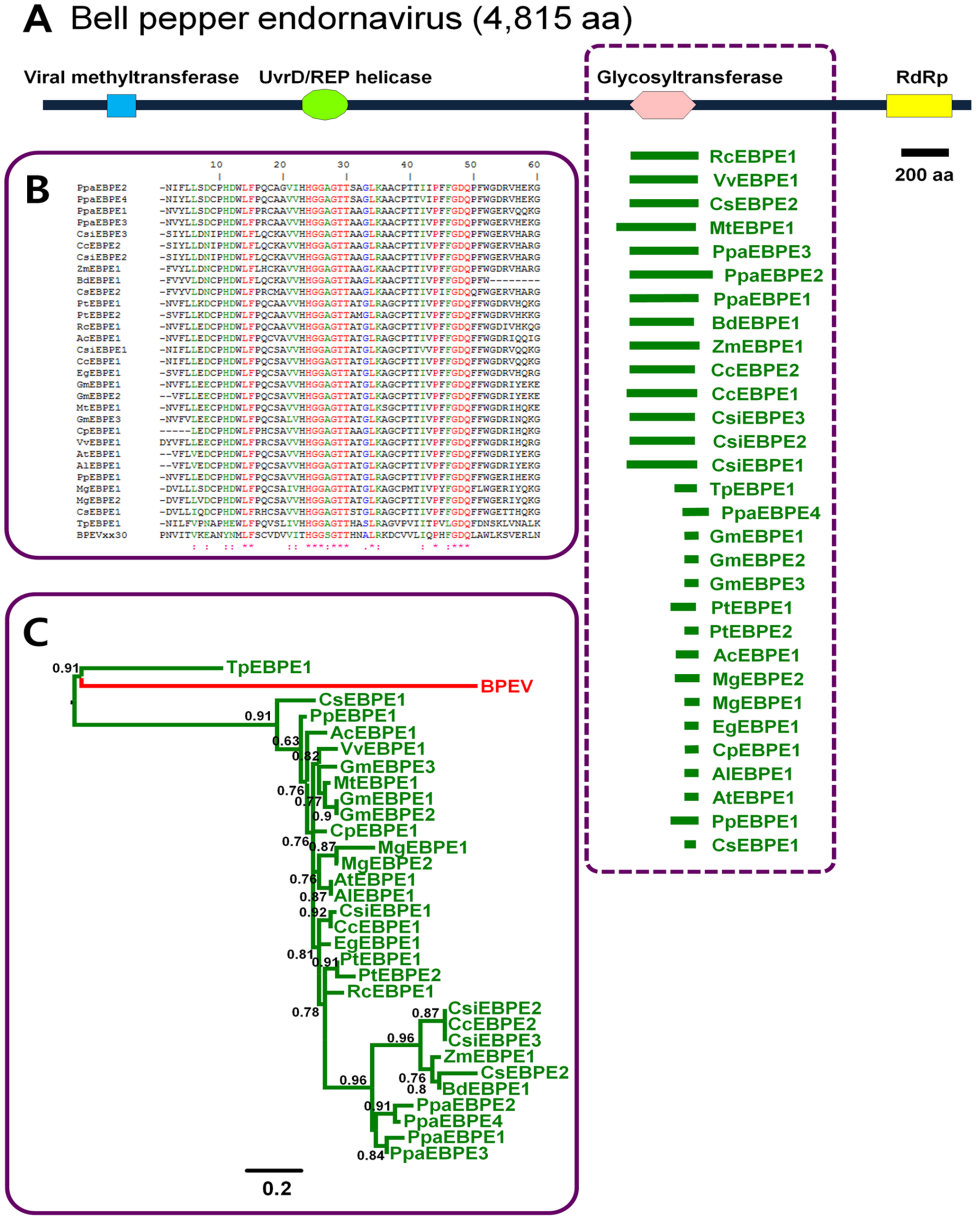
Identification of plant sequences homologous to BPEV. (A) Schematic diagram of BPEV and the corresponding locations of the plant sequences homologous to BPEV. The black bar indicates the whole proteome of BPEV, and each domain of BPEV is indicated by a symbol of a different color. The small green fragments within dotted line boxes indicate the partial plant sequences homologous to BPEV with the respective names. The abbreviated protein names can be found in Table 1. (B) Amino acid alignment of the glycosyltransferase domains of BPEV and identified plant proteins using ClustalW. (C) Phylogenetic tree based on the glycosyltransferase domains of BPEV and 30 identified plant proteins constructed using the PhyML 3.0 server. The numbers on the branches are the aLRT values calculated using a SH-like method. Numbers greater than 0.5 are shown on each branch.

**Figure 2:**
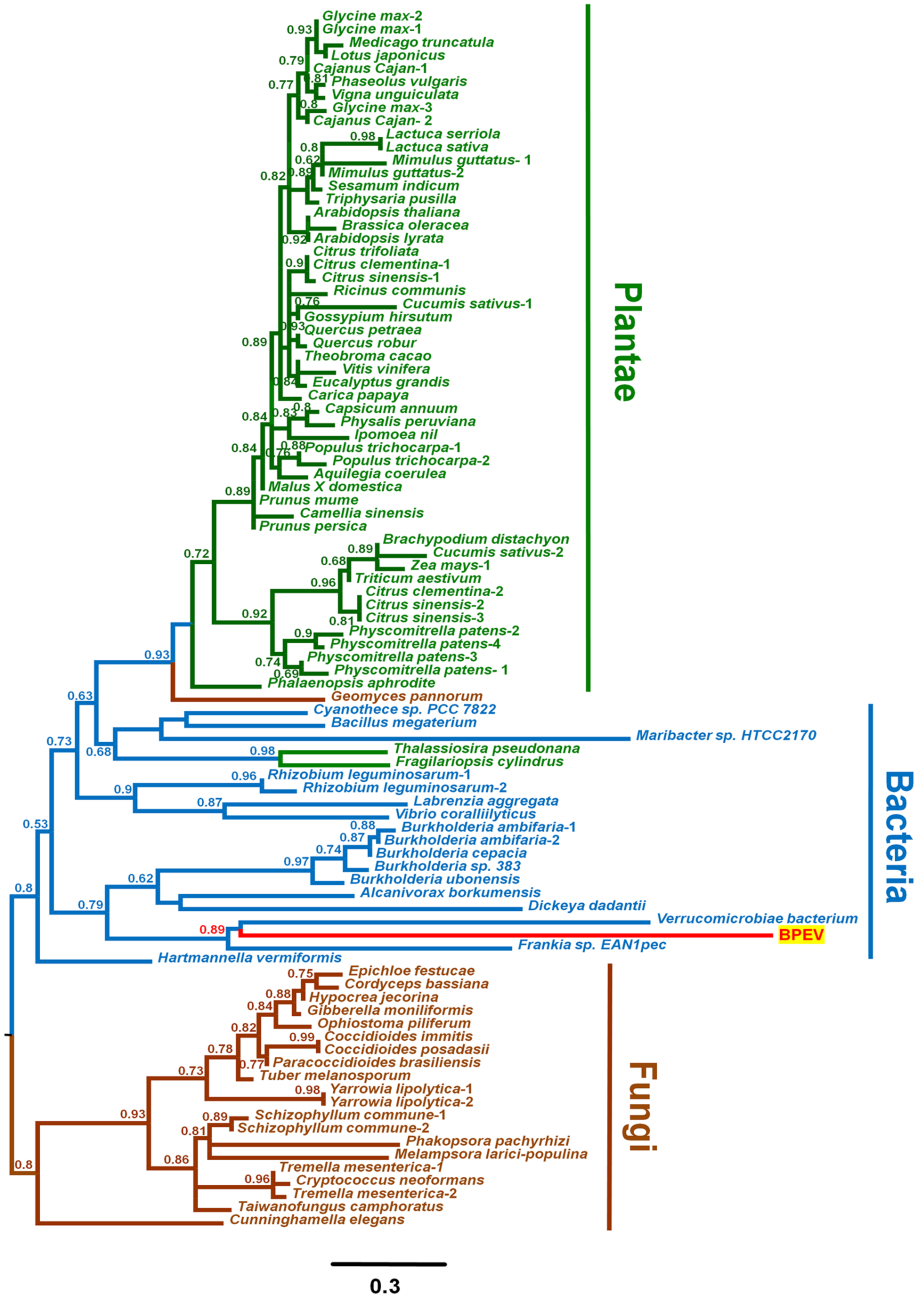
Phylogenetic relationships of the glycosyltransferase domains derived from BPEV, plants, fungi, and bacteria. The glycosyltransferase domains of various plants, fungi, and bacteria that are homologous to that of BPEV were identified by BLAST searching in several databases. The C-terminal regions of the aligned glycosyltransferase domains were used for the generation of the phylogenetic tree. A total of 93 amino acid sequences, including 54 plant sequences (in green), 21 fungal sequences (in brown), 17 bacterial sequences (in black) and the BPEV sequence (in red), were analyzed. The labels of the branches represent the aLRT values calculated using a SH-like method, and only values greater than 0.5 are displayed.

**Figure 3:**
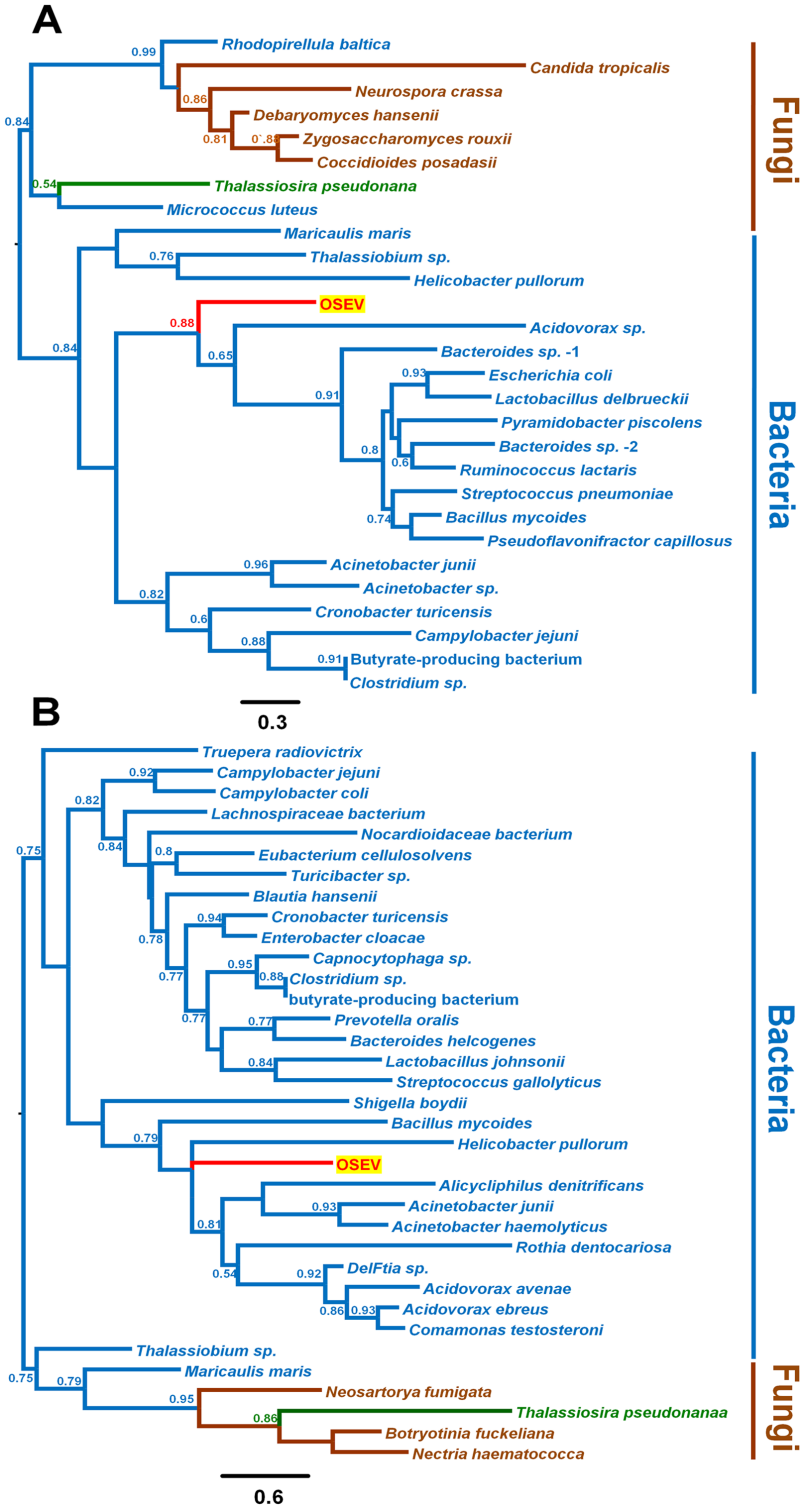
The phylogenetic relationships of two domains of OsEV and homologous proteins from other organisms. A phylogenetic tree based on the glycosyltransferase sugar-binding domain (approximately 70 amino acids) (A) and a phylogenetic tree based on the capsular polysaccharide synthesis proteins (approximately 90 amino acids) (B) derived from different organisms and OsEV. The branch colors indicate the kingdom of each organism (bacteria in black, fungi in brown, plants in green, and viruses in red). The aLRT values were calculated using a SH-like method, and values greater than 0.5 are shown on the branches.

To align and visualize sequences, ClustalW implemented in MEGA 5 software was used. The most appropriate substitution models were selected for each aligned sequence according to Akaike's information criterion (AIC) calculated using the ProtTest server (http://darwin.uvigo.es/software/prottest_server.html) [23]. For the phylogenetic analysis presented in Figure 1C, the CpREV+I+G model was selected as the best-fit substitution model. The LG+I+G model was selected for the phylogenetic analyses presented in Figure 2, Figure 3A, Figure 3B, supplementary figure S1, S3A, and S3B, each of which has a distinct gamma parameter and proportion of invariable sites. Phylogenetic trees were generated using the PhyML 3.0 server (http://www.atgc-montpellier.fr/phyml/) according to the best-fit models suggested by the ProtTest server. BIONJ was used as a starting tree, and subtree pruning and regrafting (SPR) was used for tree improvement [24]. The approximate likelihood ratio test (aLRT) values were calculated using Shimodaira-Hasegawa-like (SH-like) procedure, and each branch is labeled with the result [25]. All obtained trees were edited using FigTree version 1.3.1 (http://tree.bio.ed.ac.uk/software/).

### Detection of HGT

The 16S rRNA sequences of various species were retrieved from the SILVA rRNA database (http://www.arb-silva.de/). Phylogenetic trees based on the rRNA sequences of diverse species were generated using MEGA 5 software with the neighbor-joining method and bootstrap support of 1000 replicates after alignment using the ClustalW method. Protein trees were rerooted as species trees using FigTree version 1.3.1. The generated species and protein trees were converted into the Newick format using MEGA 5 and FigTree version 1.3.1, respectively. The detection of HGT was performed using the T-REX (Tree and Reticulogram Reconstruction) web server (http://www.trex.uqam.ca) [26].

## Results

### Identification of endornavirus-like sequences in various plant genomes

BLAST searches identified several endornavirus-sequences in plant genomes. Among sequences from known endornaviruses, only partial sequences for *Bell pepper endornavirus* (BPEV) [27] and *Oryza sativa endornavirus* (OsEV) [28] have been shown to be homologous to specific regions of plant proteins. A total of 30 non-redundant endogenous BPEV-like sequences, referred to as EBPEs, were identified in 19 plant species (Table 1). Some plant species harbor multiple EBPEs. For example, *Populus trichocarpa*, *Glycine max*, and *Cucumis sativus* each possess two EBPEs, whereas *Citrus sinensis* and *Physcomitrella patens* harbor three and four EBPEs, respectively (Table 1). Of known algae species, only *T. pseudonana* contains an EBPE, and other monocot plants, such as sorghum and rice, carry several EBPEs. Interestingly, all identified EBPEs are homologous to one specific domain of BPEV, which is referred as glycosyltransferase 28 (GT28) domain (Figure 1A). The *murG* gene of *Escherichia coli* containing the GT28 domain functions in the membrane steps of peptidoglycan synthesis [29]. The lengths of the identified EBPEs are variable, ranging from 61 to 467 amino acids (aa). The alignment of the amino acid sequences of the identified EBPEs and the GT28 domain of BPEV revealed high levels of sequence identity (%) (Figure 1B). The phylogenetic tree contains two distinct clades (Figure 1C). The first clade includes only TpEBPE and BPEV, whereas the second clade contains most EBPEs, which could be divided into two sister groups (Figure 1C).

**Table 1:** Endogenous *Bell pepper endornavirus*-like sequences (EBPEs) identified in various plants.

Index	Species Name	Database	Name	Accession No.	Identities	Evalue
1	*Ricinus communis*	Proteome	RcEBPE1	29863.m001049	78/385 (21%)	7.00E-10
2	*Populus trichocarpa*	Proteome	PtEBPE1	POPTR_0002s06770.1	38/137 (28%)	3.00E-06
3	*Populus trichocarpa*	Proteome	PtEBPE2	POPTR_0005s21520.1	24/75 (32%)	4.00E-06
4	*Medicago truncatula*	Proteome	MtEBPE1	Medtr1g125050.1	86/467 (19%)	6.00E-06
5	*Glycine max*	Proteome	GmEBPE1	Glyma20g30760.1	24/75 (32%)	2.00E-06
6	*Glycine max*	Proteome	GmEBPE2	Glyma10g36850.1	24/75 (32%)	2.00E-06
7	*Glycine max*	Proteome	GmEBPE3	Glyma02g08940.1	22/75 (30%)	3.00E-06
8	*Cucumis sativus*	Proteome	CsEBPE1	Cucsa.363720.1	21/61 (35%)	4.00E-06
9	*Cucumis sativus*	Proteome	CsEBPE2	Cucsa.356970.2	84/389 (22%)	2.00E-10
10	*Prunus persica*	Proteome	PpEBPE1	ppa002535m	38/151 (26%)	9.00E-07
11	*Arabidopsis thaliana*	Proteome	AtEBPE1	AT1G43620.1	24/75 (32%)	9.00E-07
12	*Arabidopsis lyrata*	Proteome	AlEBPE1	473768	24/75 (32%)	7.00E-07
13	*Carica papaya*	Proteome	CpEBPE1	evm.model.supercontig_21.209	21/71 (30%)	9.00E-06
14	*Citrus sinensis*	Proteome	CsiEBPE1	orange1.1g006412m	75/394 (20%)	4.00E-07
15	*Citrus sinensis*	Proteome	CsiEBPE2	orange1.1g013835m	78/361 (22%)	1.00E-08
16	*Citrus sinensis*	Proteome	CsiEBPE3	orange1.1g013358m	80/364 (22%)	2.00E-08
17	*Citrus clementina*	Proteome	CcEBPE1	clementine0.9_004746m	76/394 (20%)	2.00E-07
18	*Citrus clementina*	Proteome	CcEBPE2	clementine0.9_010298m	80/364 (22%)	2.00E-08
19	*Eucalyptus grandis*	Proteome	EgEBPE1	Egrandis_v1_0.005809m	23/75 (31%)	4.00E-06
20	*Vitis vinifera*	Proteome	VvEBPE1	GSVIVT01034544001	81/387 (21%)	3.00E-08
21	*Mimulus guttatus*	Proteome	MgEBPE1	mgv1a022288m	21/80 (27%)	9.00E-07
22	*Mimulus guttatus*	Proteome	MgEBPE2	mgv1a010079m	38/131 (30%)	4.00E-06
23	*Aquilegia coerulea*	Proteome	AcEBPE1	AcoGoldSmith_v1.003466m	36/123 (30%)	1.00E-06
24	*Zea mays*	Proteome	ZmEBPE1	GRMZM2G007721_T01	86/395 (22%)	7.00E-08
25	*Brachypodium distachyon*	Proteome	BdEBPE1	Bradi5g01230.1	81/359 (23%)	1.00E-08
26	*Physcomitrella patens*	Proteome	PpaEBPE1	Pp1s110_116V6.1	83/388 (22%)	2.00E-09
27	*Physcomitrella patens*	Proteome	PpaEBPE2	Pp1s245_59V6.1	97/463 (21%)	4.00E-09
28	*Physcomitrella patens*	Proteome	PpaEBPE3	Pp1s159_62V6.1	84/388 (22%)	4.00E-11
29	*Physcomitrella patens*	Proteome	PpaEBPE4	Pp1s281_20V6.1	38/142 (27%)	7.00E-06
30	*Thalassiosira pseudonana*	Proteome	TpEBPE1	269844	42/132 (32%)	2.00E-07

The accession number of each protein can be found in the peptide data for the individual plant species deposited in Phytozome. Abbreviation: endogenous *Bell pepper endornavirus*-like sequences (EBPEs). Each identified protein is named after the plant species. For example, EBPE1 from *Ricinus communis* is referred to as RcEBPE1. All identified EBPEs in plants are homologous to the sequence of the glycosyltransferase (GT) 28 domain in *Bell pepper endornavirus* (Accession No. NC_015781).

### Identification of EBPEs from various databases

With the development of several high-throughput sequencing technologies, a large number of sequencing data from many plant species are being produced [30]. We also identified 29 EBPEs from expressed sequence tag (EST) (18 sequences) and transcriptome shotgun assembly (TSA) (9 sequences) databases (Table 2). Of these 29 EBPEs, one was identified among the ESTs of the pepper plant (*Capsicum annuum*), which is a host plant for BPEV. In a search for EBPEs in various databases, we found that the GT28 domain exists in other organisms, including bacteria and fungi (Table 3). For example, 21 EBPEs were derived from 18 fungal species including *Cryptococcus neoformans*, *Coccidioides immitis*, and *Coccidioides posadasii*, and these fungi each possess a GT28 domain with 30% to 43% identity to the GT28 domain BPEV (Table 3). In addition, many EBPEs are present in diverse bacteria, including *Verrucomicrobiae bacterium*, *Burkholderia ambifaria*, and *Burkholderia ubonensis*. Rather than identifying EBPEs using BLAST searches, we identified sequences containing the GT28 domain (PF03033) in the Pfam database (http://pfam.sanger.ac.uk/) [31]. We found 2,898 sequences containing the GT28 domain in 2,051 species. Most of these sequences are derived from various bacteria (2,636 sequences from 1,963 species). Only 251 sequences are derived from the Eukaryota, covering 89 species, and 7 sequences are from the Archaea, covering 4 species belonging to the family *Methanosarcinaceae* (Table 3).

**Table 2:** Endogenous EBPEs identified in EST and TSA databases.

Index	Genus	Database	Name	Accession No.	Identities	Evalue
1	*Phaseolus vulgaris*	EST	PvEBPE1	GW903724.1	76/135 (56%)	3.00E-44
2	*Phaseolus vulgaris*	EST	PvEBPE2	GW910780.1	66/118 (56%)	4.00E-36
3	*Phaseolus vulgaris*	EST	PvEBPE3	GW886643.1	55/105 (52%)	1.00E-27
4	*Phaseolus vulgaris*	EST	PvEBPE4	GW897466.1	54/105 (51%)	5.00E-27
5	*Phaseolus vulgaris*	EST	PvEBPE5	FE705619.1	24/75 (32%)	2.00E-05
6	*Gossypium hirsutum*	EST	GhEBPE1	AI729743.1	25/80 (31%)	7.00E-07
7	*Quercus petraea*	EST	QpEBPE1	FP047005.1	25/75 (33%)	5.00E-06
8	*Quercus robur*	EST	QrEBPE1	FR632001.1	24/72 (33%)	2.00E-05
9	*Prunus mume*	EST	PmEBPE1	GW871929.1	24/75 (32%)	6.00E-06
10	*Brassica oleracea*	EST	BoEBPE1	EE530426.1	24/75 (32%)	6.00E-06
11	*Malus X domestica*	EST	MdEBPE1	GO512845.1	24/75 (32%)	1.00E-05
12	*Citrus trifoliate*	EST	CtEBPE1	CV707023.1	23/75 (31%)	1.00E-05
13	*Lotus japonicas*	EST	LjEBPE1	GO012114.1	24/75 (32%)	2.00E-05
14	*Theobroma cacao*	EST	TcEBPE1	CU573005.1	24/75 (32%)	2.00E-05
15	*Capsicum annuum*	EST	CaEBPE1	GD079868.1	26/80 (33%)	2.00E-05
16	*Triphysaria pusilla*	EST	TpuEBPE1	EY149399.1	23/75 (31%)	3.00E-05
17	*Ipomoea nil*	EST	InEBPE1	BJ568418.1	26/79 (33%)	4.00E-05
18	*Vigna unguiculata*	EST	VuEBPE1	FG925725.1	23/75 (31%)	6.00E-05
19	*Fragilariopsis cylindrus*	EST	FcEBPE1	GW068715.1	28/63 (44%)	1.00E-05
20	*Hartmannella vermiformis*	EST	HvEBPE1	EC129829.1	28/88 (32%)	1.00E-05
21	*Cajanus cajan*	TSA	CcEBPE1	EZ628202.1	24/75 (32%)	8.00E-07
22	*Cajanus cajan*	TSA	CcEBPE2	EZ652459.1	22/75 (29%)	7.00E-06
23	*Camellia sinensis*	TSA	CasEBPE1	HP735048.1	24/82 (29%)	5.00E-06
24	*Sesamumindicum*	TSA	SiEBPE1	JL373686.1	22/75 (29%)	1.00E-05
25	*Physalis peruviana*	TSA	PpeEBPE1	JO132297.1	26/80 (33%)	1.00E-05
26	*Phalaenopsis aphrodite*	TSA	PhaEBPE1	JI639559.1	21/75 (28%)	5.00E-05
27	*Lactuca serriola*	TSA	LsEBPE1	JO026119.1	24/78 (31%)	5.00E-05
28	*Lactuca sativa*	TSA	LsaEBPE1	JI575620.1	24/78 (31%)	5.00E-05
29	*Triticum aestivum*	TSA	TaEBPE1	HP636920.1	24/78 (31%)	9.00E-05

Abbreviations: expressed sequence tag (EST), transcriptome shotgun assembly (TSA).

The accession number of each identified gene can be used to retrieve sequences from the EST and TSA databases of NCBI.

**Table 3:** Endogenous EBPEs identified in fungi and bacteria.

Index	Genus	Database	Group	Name	Accession No.	Identities	Evalue
1	*Cryptococcus neoformans*	EST	Fungi	CnEBPE1	CF697842.1	27/77 (35%)	1.00E-07
2	*Coccidioides immitis*	EST	Fungi	CiEBPE1	GH386888.1	31/86 (36%)	1.00E-06
3	*Coccidioides posadasii*	EST	Fungi	CpoEBPE1	GH450130.1	29/68 (43%)	2.00E-06
4	*Gibberella moniliformis*	EST	Fungi	GmoEBPE1	DR644897.1	27/66 (41%)	1.00E-06
5	*Taiwanofungus camphoratus*	EST	Fungi	TcaEBPE1	DR027260.1	26/82 (32%)	1.00E-06
6	*Ophiostoma piliferum*	EST	Fungi	OpEBPE1	EB042199.1	27/67 (40%)	2.00E-06
7	*Tremella mesenterica*	EST	Fungi	TmEBPE1	GR248393.1	25/65 (38%)	2.00E-06
8	*Tremella mesenterica*	EST	Fungi	TmEBPE2	GR229188.1	24/65 (37%)	2.00E-05
9	*Phakopsora pachyrhizi*	EST	Fungi	PhpEBPE1	EH227244.1	25/80 (31%)	2.00E-06
10	*Tuber melanosporum*	EST	Fungi	TmeEBPE1	FP395975.1	27/81 (33%)	3.00E-06
11	*Yarrowia lipolytica*	EST	Fungi	YlEBPE1	FP686306.1	28/79 (35%)	9.00E-06
12	*Yarrowia lipolytica*	EST	Fungi	YlEBPE2	FP688580.1	25/66 (38%)	4.00E-05
13	*Cordyceps bassiana*	EST	Fungi	CbEBPE1	DT370188.1	25/63 (40%)	1.00E-05
14	*Hypocrea jecorina*	EST	Fungi	HjEBPE1	CF867280.1	25/63 (40%)	1.00E-05
15	*Melampsora larici-populina*	EST	Fungi	MlEBPE1	GR774815.1	25/62 (40%)	2.00E-05
16	*Epichloe festucae*	EST	Fungi	EfEBPE1	GO828487.1	25/69 (36%)	2.00E-05
17	*Paracoccidioides brasiliensis*	EST	Fungi	PbEBPE1	CN244888.1	25/62 (40%)	2.00E-05
18	*Schizophyllum commune*	EST	Fungi	ScEBPE1	GW366092.1	24/81 (30%)	4.00E-05
19	*Schizophyllum commune*	EST	Fungi	ScEBPE2	GW362507.1	24/72 (33%)	8.00E-05
20	*Geomyces pannorum*	EST	Fungi	GpEBPE1	DY989210.1	24/62 (39%)	8.00E-05
21	*Cunninghamella elegans*	EST	Fungi	CeEBPE1	DY894586.1	24/80 (30%)	8.00E-05
22	*Verrucomicrobiae bacterium*	Genome	Bacteria	VbEBPE1	ABSI01000010.1	25/69 (36%)	1.00E-06
23	*Burkholderia ambifaria*	Genome	Bacteria	BaEBPE1	ABLK01000280.1	25/58 (43%)	1.00E-06
24	*Burkholderia ambifaria*	Genome	Bacteria	BaEBPE2	ABLC01000025.1	23/58 (40%)	2.00E-05
25	*Burkholderia ubonensis*	Genome	Bacteria	BuEBPE1	ABBE01000023.1	24/63 (38%)	5.00E-06
26	*Burkholderia sp. 383*	Genome	Bacteria	BspEBPE1	NC_007511.1	23/58 (40%)	3.00E-05
27	*Burkholderia cepacia*	Genome	Bacteria	BcEBPE1	NC_008391.1	22/58 (38%)	4.00E-05
28	*Alcanivorax borkumensis*	Genome	Bacteria	AbEBPE1	NC_008260.1	25/79 (32%)	1.00E-06
29	*Labrenzia aggregata*	Genome	Bacteria	LaEBPE1	AAUW01000001.1	30/91 (33%)	5.00E-06
30	*Rhizobium leguminosarum*	Genome	Bacteria	RlEBPE1	NC_012853.1	29/64 (45%)	5.00E-06
31	*Rhizobium leguminosarum*	Genome	Bacteria	RlEBPE2	NC_008384.1	27/64 (42%)	4.00E-05
32	*Frankia sp. EAN1pec*	Genome	Bacteria	FspEBPE1	NC_009921.1	26/61 (43%)	6.00E-06
33	*Bacillus megaterium*	Genome	Bacteria	BmEBPE1	NC_014019.1	25/85 (29%)	9.00E-06
34	*Maribacter sp. HTCC2170*	Genome	Bacteria	MsEBPE1	NC_014472.1	27/73 (37%)	1.00E-05
35	*Vibrio coralliilyticus*	Genome	Bacteria	VcEBPE1	ACZN01000014.1	28/79 (35%)	8.00E-05
36	*Cyanothece sp. PCC 7822*	Genome	Bacteria	CspEBPE1	NC_014533.1	28/92 (30%)	8.00E-05
37	*Dickeya dadantii*	Genome	Bacteria	DdEBPE1	NC_013592.1	27/69 (39%)	8.00E-05

### Phylogenetic relationships and domain structures for plant GT28-containing proteins

BLAST searches using the BPEV sequences could not detect all GT28-containing proteins in plant genomes. Using the Phytozome ver. 7 database (http://www.phytozome.net) [32], we found 78 proteins containing the GT28 domain from 23 plant species. To elucidate the phylogenetic relationships of GT28-containing proteins, we constructed a phylogenetic tree (Figure S1A). The phylogenetic tree shows the most GT28-containing plant proteins except CsEBPE1 are closely related. The domain structures of GT28-containing proteins show the localization of the GT28 domain in each protein (Figure S1B). Most GT28 domains are located in the 5′ region of each protein, but three EBPEs from *Physcomitrella patens* have the GT28 domain in the middle of the protein (Supplementary figure S1B). Interestingly, most GT28-containing proteins include an additional domain referred to as a UDP glycosyltransferase (UGT) (PF0001) in the C-terminal region. Algae including *Chlamydomonas reinhardtii* and *Volvox carteri* encode proteins containing only a GT28 domain and not a UGT domain, whereas mosses including *Physcomitrella patens* and *Selaginella moellendorffii* have several proteins that contain both the GT28 and UGT domains. In addition, the numbers of exons and introns in GT28-containing genes in plants are diverse. For example, two *Arabidopsis thaliana* genes containing the GT28 domain consist of 15 exons and 14 introns whereas two *Arabidopsis lyrata* genes containing GT28 domain comprise 14 exons and 13 introns.

### Phylogenetic relationships of all identified EBPEs

To reveal the phylogenetic relationships of EBPEs and the origin of the GT28 domain in BPEV, we constructed a phylogenetic tree using all identified EBPEs from plants, bacteria, and fungi. The phylogenetic tree includes two largely divided clades (Figure 2). The first clade encompasses EBPEs from plants, fungi, and bacteria, whereas the second clade comprises only EBPEs from fungi. In the first clade, the plant EBPEs appear to be generally monophyletic except for those of two diatoms, *T. pseudonana* and *Fragilariopsis cylindrus*. Interestingly, the clade containing these two diatoms includes three bacteria species, *Cyanothece* sp. PCC 7822, *Bacillus megaterium*, and *Maribacter* sp. HTCC2170. Surprisingly, the GT28 of BPEV is closely related to those of two bacteria, *Verrucomicrobiae bacterium* and *Frankia* sp. EAN1pec. In addition, *Geomyces pannorum*, which is a type of saprophytic fungi, is grouped together with other higher plants. These results suggest that the gene transfer of the GT20 domain might have occurred among diatoms, bacteria, fungi, and endornaviruses.

### Conserved domains present in nine endornaviruses

Based on the above result, we hypothesized that other domains present in endornaviruses might have originated from other organisms. Next, we examined the conserved domains of nine endornaviruses for which the complete protein sequences are currently available (Supplementary figure S2). The ORF lengths of these endornaviruses ranges from 3,217 aa to 5,825 aa. The *Vicia faba endornavirus* (VfEV) is the largest endornavirus, but it has only two conserved domains, a viral helicase domain, and an RdRp. *Tuber aestivum* endornavirus (TaEV) is the smallest endornavirus containing a DEAD-like helicase domain and an RdRp. Although all nine endornaviruses contain an RdRp domain, the compositions of the other domains are highly variable. One of the common domains in the nine endornaviruses is the UGT domain, which is present in six endornaviruses that infect plants, fungi or protists (Supplementary figure S2). No sequences in plants highly similar to the UGT sequence were identified. In addition, there is no available information for UGT in the Pfam database.

### Phylogenetic analysis of two distinct glycome-related domains in OsEV

In addition to UGT, OsEV contains two glycome-related domains, the glycosyltransferase sugar-binding region containing DXD motif (GTS) (PF04488) and the capsular polysaccharide synthesis protein (CPSP) (PF05704) (Supplementary figure S2). GTS is a GT, and the DXD motif of GTS is required for carbohydrate binding in sugar-nucleoside diphosphate- and manganese-dependent glycosyltransferases [33]. According to the Pfam database, there are at least 508 species, including 175 Eukaryota, 326 bacteria, 5 viruses, and 1 Archaea, that encode the GTS domain. To identify the phylogenetic relationships of GTSs from various species, we performed a BLAST search and constructed a phylogenetic tree, which contains two distinct clades (Figure 3A). The first clade includes only GTSs from diverse bacteria and OsEV. The second clade is composed of primarily of GTSs from fungi, along with one diatom (*T. pseudonana*) and two bacteria (*Rhodopirellula baltica* and *Micrococcus luteus*). The GTS of *T. pseudonana* is more closely related to that of *M. luteus*. Next, we searched the Pfam database and identified 235 sequences from 171 species containing CPSP; these species included 163 bacteria, 25 Eukaryota, and one virus. Using the CPSP sequences highly homologous to that of OsEV, we constructed a phylogenetic tree, which had two distinct clades (Figure 3B). The first clade contained sequences from OsEV and bacteria. The second clade included *T. pseudonana,* three fungi (*Neosartorya fumigata*, *Botryotinia fuckeliana*, and *Nectria haematococca*), and two bacteria (*Thalassibium* sp. and *Maricaulis maris*).

### Phylogenetic analysis using RdRp domains of endornaviruses

All endornaviruses have an RdRp (PF00978), which catalyzes the replication of RNA from an RNA template. According to the Pfam database, only viruses (427 species) possess RdRp domains (PF00978). To find possible origin of the RdRp in the genus *Endornavirus*, we first collected sequences highly homologous to that of the RdRp of BPEV, and most of these sequences were derived from other endornaviruses and single-stranded (ss) RNA viruses. The phylogenetic tree based on the RdRp sequences includes two distinct clades (Supplementary figure S3A). The first clade consists of solely endornaviruses along with two different ssRNA viruses, *Lilac Ring Mottle virus* and *Apple stem pitting virus*, whereas the second clade contains solely ssRNA viruses.

Two endornaviruses, GaBRV-XL and TaEV, that infect fungi contain the DEAD box helicase (DEXDc) domain (PF00270). A total of 3,526 species, including 2,661 bacteria, 561 Eukaryota, 142 Archaea, and 200 viruses, possess a DEXDc domain according to the PFAM database. A phylogenetic tree was constructed using the sequences highly homologous to the DEXDc sequences of GaBRV-XL and TaEV, and this tree contains two clades (Supplementary figure S3B). The first clade contains various bacteria in addition to two endornaviruses and one fungus (*Sclerotinia sclerotiorum*). Two other viruses, *Modoc virus* and *Simian varicella virus*, belong to the first clade. In contrast, the second clade consists of various organisms including green algae, protozoa, and Archaea.

### Prediction of horizontal gene transfer for each domain in endornaviruses

**Figure 4:**
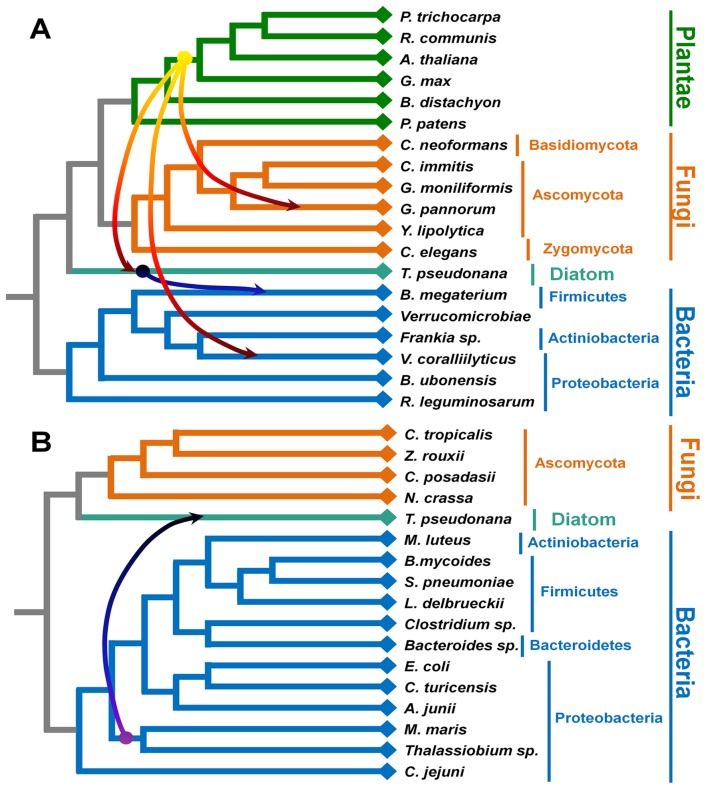
Horizontal gene transfer of two domains, (A) the glycosyltransferase 28 domain and (B) the glycosyltransferase sugar-binding region, among three different kingdoms. The amino acid sequences for the glycosyltransferase 28 domain and the glycosyltransferase sugar-binding region and the corresponding rRNA sequences from the various species representing three different kingdoms (plants in green, fungi in brown, and bacteria in blue) were used. Predicted HGTs are represented by arrows from the original species to the recipient species. The detection of HGT was based on the 16S rRNA sequences of various species.

The phylogenetic analyses suggested that at least BPEV and OsEV acquired several domains via HGT. It is likely that HGT of glycome-related domains might have occurred among different organisms. To assess this possibility, we compared trees between given pairs of species and domains as described in the materials and methods. We excluded endornaviruses from the analyses, as these are not assigned to the tree of life. In the case of the GT28 domain, at least four independent HGTs have occurred among plants, fungi, and bacteria (Figure 4A). The GT28 domain in plants might have been transferred to *Geomyces pannorum*, *T. pseudonana*, and *Vibrio coralliilyticus*. The *Bacillus megaterium* obtained GT28 domain from *T. pseudonana*. The GTSs of two proteobacteria, *M. maris* and *Thalassiobium* sp., might have been horizontally transferred to the genome of *T. pseudonana* (Figure 4B).

## Discussion

In the current study, we conducted phylogenetic analyses to explore the evolutionary origins of protein domains present in endornaviruses. Due to the limited number of available sequences for endornaviruses, only a few domains for endornaviruses were further analyzed. Our analyses allowed us to (i) identify endornavirus-like sequences in plants, fungi, and bacteria, (ii) reveal the phylogenetic relationships among these sequences, and (iii) elucidate the evolutionary origins of endornaviral genes by HGT.

Initially, all available endornavirus sequences were used in BLAST searches to identify endornavirus-like sequences in plant proteomes. Only partial sequences for BPEV and OsEV were matched to various plant proteomes, indicating that gene transfer might have occurred between endornaviruses and plant hosts. An extensive BLAST search and domain information from the Pfam database revealed that three domains, the GT28, GTS, and CPSP domains, are ubiquitous; these domains are present in Eukaryota, bacteria, Archaea, and viruses. These results suggest that some endornaviral genes might have been obtained from the host or transferred from other organisms by HGT. Phylogenetic analyses demonstrated that the GT28 domain of BPEV is highly homologous to those of some bacteria, suggesting a possible origin of the GT28 domain in BPEV. Three bacteria, *Cyanothece* sp. PCC 7822, *Bacillus megaterium*, and *Maribacter* sp. HTCC2170, as well as *T. pseudonana* are present together with BPEV in the same clade, and all three live in marine and freshwater environments. This result suggests two possible scenarios for how endornaviruses acquired the GT28 domain from their hosts or other organisms. The first scenario is direct horizontal gene transfer from marine bacteria to ancient endornaviruses that infect marine algae such as diatoms via unknown events, which have caused genetic recombination. The second scenario is that marine diatoms first obtained the GT28 domain from marine bacteria that infect the diatoms, and then the ancient endornaviruses obtained the GT28 domain from the marine diatom host. *T. pseudonana* is a marine diatom that acquires plastids through secondary endosymbiosis [34]; a previous study found that *T. pseudonana* has acquired foreign genes such as membrane transporter genes via endosymbiotic/horizontal gene transfer (E/HGT) to adapt them in marine environments [35]. Moreover, a recent study suggested that *T. pseudonana* is likely ancestrally a freshwater organism [36]. Therefore, we tentatively support the second scenario because the HGT of the GT28 gene could have occurred between diatoms and bacteria due to their presence in marine and freshwater environments and because phylogenetic evidence revealed that the sequences for *T. pseudonana* and BPEV were in the same clade. To date, endornaviruses have been identified only in Eukaryota, including plants, fungi, and Chromista [22]. Based on our analysis, we propose the existence of endornaviruses that infect marine algae. The ancient endornaviruses that infected marine algae might have co-evolved with their hosts, and they might have begun infecting land plants during the evolution of higher plants. Thus, unidentified endornaviruses that infect marine algae have domain structures that are very similar to those of endornaviruses that infect higher plants. It is known that endornaviruses are only vertically transmitted through seeds [22], which could support the co-evolution of the endornavirus with their hosts.

The *Arabidopsis* genome contains two genes (*UGT80A2* and *UGT80B1*) that possess GT28 and UGT domains; these genes encode UDP-glucose:sterol glycosyltransferases enzymes (EC 2.4.1.173) [37]. These enzymes are involved in the synthesis of steryl glycosides (SGs) [38]. The *UGT80A2* mutant showed mild defects in plant growth, whereas the *UGT80B1* mutant exhibited severe phenotypes at both the embryo and seed stages. *UGT80B1* is required for the deposition of flavanoids, the suberization of the seed, and the trafficking of lipid polyesters in membranes [37]. Genes encoding SGs are ubiquitous in plants [39] and various fungi [40]. The null mutant of this gene in *Saccharomyces cerevisiae* exhibited normal growth under diverse culture conditions despite the reduced ability to synthesize sterol glucoside [40]. In bacteria, the *murG* gene from *Escherichia coli* contains two duplicated GT20 domains localized at the N-terminal (PF03033) and C-terminal (PF04101) regions, respectively. The mutation of *murG* led to an altered cell shape and a lytic thermosensitive phenotype [29]. CPSP is known to be a major virulence factor in *Streptococcus pneumonia* and plays an important role in the production of a mature capsule *in vitro* [41]. Thus, the functions of genes related to glycosyltransferases are diverse and vary depending on the organism.

It is known that some DNA viruses contain several GT domains, but hypoviruses are the first RNA viruses known to encode GTs named as UGT [42], [43]. It is also likely that the UGTs of hypoviruses might be originated from the host genes. Endornaviruses have at least three different domains that are highly associated with glycome modification [22]. Interestingly, all three are closely related to those of marine bacteria. The GTs encoded by DNA viruses have been well characterized. Bacteriophages, phycodnaviruses, baculoviruses, poxviruses, and herpesviruses contain genes encoding GTs [44]. These DNA viruses appear to have co-evolved with their hosts, and they acquired the GTs for replication [44]. The glycome plays an important role in many biological processes. For instance, the viral GTs of DNA viruses are involved in many different mechanisms, such as the recognition of host cells and the regulation of virus-host interactions, which are regulated by the expression of host GTs or their own viral GTs [45], [46]. Moreover, the GTs of some DNA viruses play a role is disrupting host defense mechanisms by inhibiting the activities of host restriction enzymes [44]. However, nothing is known about the functional roles of GTs in endornaviruses. It will be of interest to elucidate the functions of GTs in RNA viruses in the future. Based on the previous study, the function of GTs in endornaviruses should be beneficial to the virus [44]. As suggested in the previous study, GTs in endornaviruses might function in protection of the viral RNA from degradation by modifying the RNA [22].

Previous several studies also suggested that many viral genes might have originated from prokaryotic or eukaryotic genes [47]. For example, the heat shock protein 70 in the family *Closteroviridae*, AlkB protein in the family *Flexiviridae*, and Maf/HAM1-likepyrophosphatase in the family *Potyviridae* are originated from the host genes via horizontal gene transfer [48]–[50]. In general, they are ubiquitous genes presenting in prokaryotes and eukaryotes, and play important roles in viral life cycles [47].

All endornaviruses contain a well-conserved RdRp, and some endornaviruses contain methyltransferase and helicase domains like most RNA viruses do. As shown in our study and in a previous study, the RdRp and viral methyltransferases of endornaviruses are similar to those of ssRNA viruses [22]. These data suggest that endornaviruses might have originated from ssRNA viruses or that the important domains in endornaviruses might have been obtained from ssRNA viruses via HGT [20]. In addition, a phylogenetic analysis found that the DEXDc domains present in GaBRV-XL and TaEV are closely related to those of bacteria and that of the fungus known as *Sclerotinia sclerotiorum*. Interestingly, a hypovirulent double-stranded (ds) RNA virus has been previously identified in the plant pathogen *Sclerotinia sclerotiorum* [51]. Therefore, we tentatively hypothesize that gene transfer occurred between the fungal host and dsRNA mycoviruses. Recently, several studies have confirmed that horizontal gene transfer has occurred between mycoviruses and the host [52].

In summary, we provide strong evidence for the HGT of domains present in endornaviruses, and we proposed hypotheses regarding their possible origin and the evolutionary scenario using phylogenetic data. Although several recent studies provide evidence for HGT, gene transfer between the virus and the host is still poorly understood. To elucidate the origin and the evolutionary processes of viral genes, rigorous systematic studies, including comparative sequence analyses and experimental studies, should be conducted.

## Supporting Information

Figure S1 **Phylogenetic relationships among plant proteins containing the glycosyltransferase 28 domain.** (A) A phylogenetic tree was constructed based on proteins containing the glycosyltransferase 28 domain from 23 plant species. The aLRT values of each branch were calculated using a SH-like method, and values greater than 0.5 are shown. (B) The relative size of each plant protein is illustrated by the black bar. The schematic localization of the glycosyltransferase 28 domain in each plant protein is illustrated with yellow boxes.
(TIF)

Figure S2 **Schematic diagrams of the polyprotein structures for the 11 endornaviruses whose whole genome sequences have been determined.** Each domain is indicated by a symbol of a different color with a description below the domain. Abbreviations: *Bell pepper endornavirus*, BPEV; *Oryza sativa endornavirus*, OsEV; *Oryza rufipogon endornavirus*, OREV; *Vicia faba endornavirus*, VfEV; *Gremmeniella abietina* type B RNA virus XL, GaBRV-XL; *Tuber aestivum* endornavirus, TaEV; *Chalara elegans* endornavirus 1, CeEV1; *Phytophthora endornavirus 1*, PEV1; *Helicobasidium mompa endornavirus 1*, HmEV; RNA-dependent RNA polymerase, RdRp; glycosyltransferase sugar-binding domain, Glycosyltransfer-sug; capsular polysaccharide synthesis protein, Caps synth; DEAD box helicase, DEXDc. The scale bar at the bottom represents the relative length of the amino acid sequence.
(TIF)

Figure S3 **Phylogenetic relationships of endornaviruses and RNA viruses based on the RdRp and the helicase domain.** (A) A phylogenetic tree was constructed based on the RdRp sequences of 15 endornaviruses and 15 RNA viruses. The sequences of only the RNA viruses whose RdRp sequences were highly similar to those of endornaviruses were selected. The red and black colors indicate endornaviruses and RNA viruses, respectively. (B) A phylogenetic tree was constructed based on DEAD-like helicase (DEXDc) domains derived from bacteria (in blue), endornaviruses (in red), a fungus (in brown), and other viruses (in black). Amino acid sequences highly homologous to the DEXDc sequences of three endornaviruses were used for the phylogenetic analysis. The aLRT values of each branch were calculated using a SH-like method, and values greater than 0.5 are shown. SVV is an abbreviation for *Simian varicella virus*.
(TIF)
