# Supplementary figures and images for: Evolution of and Horizontal Gene Transfer in the Endornavirus Genus
Source: PLoS One. 2013 May 7;8(5):e64270. doi: 10.1371/journal.pone.0064270 (PMC3647011; doi:10.1371/journal.pone.0064270)

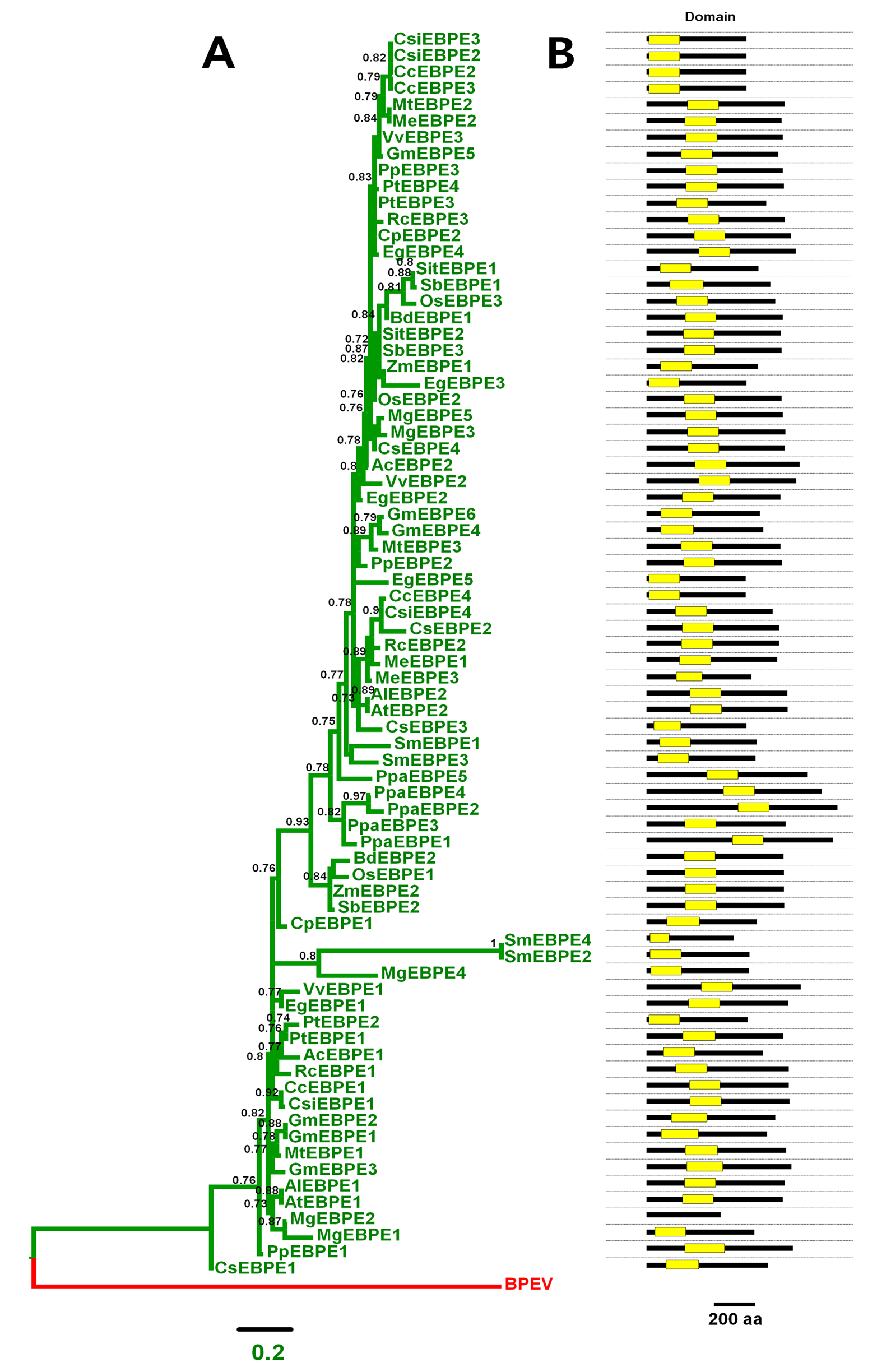

Supplement: Figure S1 — Phylogenetic relationships among plant proteins containing the glycosyltransferase 28 domain. (A) A phylogenetic tree was constructed based on proteins containing the glycosyltransferase 28 domain from 23 plant species. The aLRT values of each branch were calculated using a SH-like method, and values greater than 0.5 are shown. (B) The relative size of each plant protein is illustrated by the black bar. The schematic localization of the glycosyltransferase 28 domain in each plant protein is illustrated with yellow boxes. (TIF) [file pone.0064270.s001.tif]

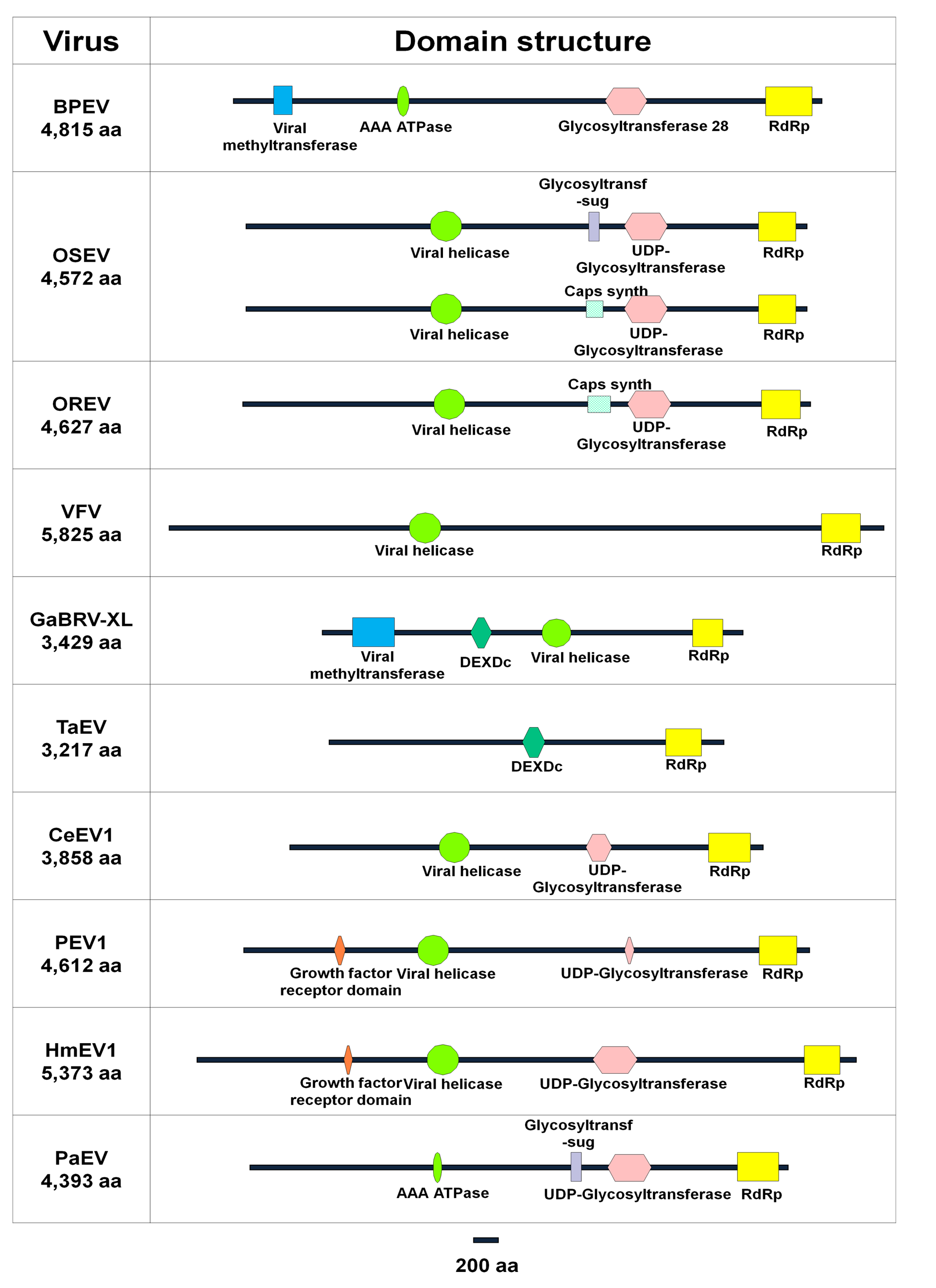

Supplement: Figure S2 — Schematic diagrams of the polyprotein structures for the 11 endornaviruses whose whole genome sequences have been determined. Each domain is indicated by a symbol of a different color with a description below the domain. Abbreviations: Bell pepper endornavirus, BPEV; Oryza sativa endornavirus, OsEV; Oryza rufipogon endornavirus, OREV; Vicia faba endornavirus, VfEV; Gremmeniella abietina type B RNA virus XL, GaBRV-XL; Tuber aestivum endornavirus, TaEV; Chalara elegans endornavirus 1, CeEV1; Phytophthora endornavirus 1, PEV1; Helicobasidium mompa endornavirus 1, HmEV; RNA-dependent RNA polymerase, RdRp; glycosyltransferase sugar-binding domain, Glycosyltransfer-sug; capsular polysaccharide synthesis protein, Caps synth; DEAD box helicase, DEXDc. The scale bar at the bottom represents the relative length of the amino acid sequence. (TIF) [file pone.0064270.s002.tif]

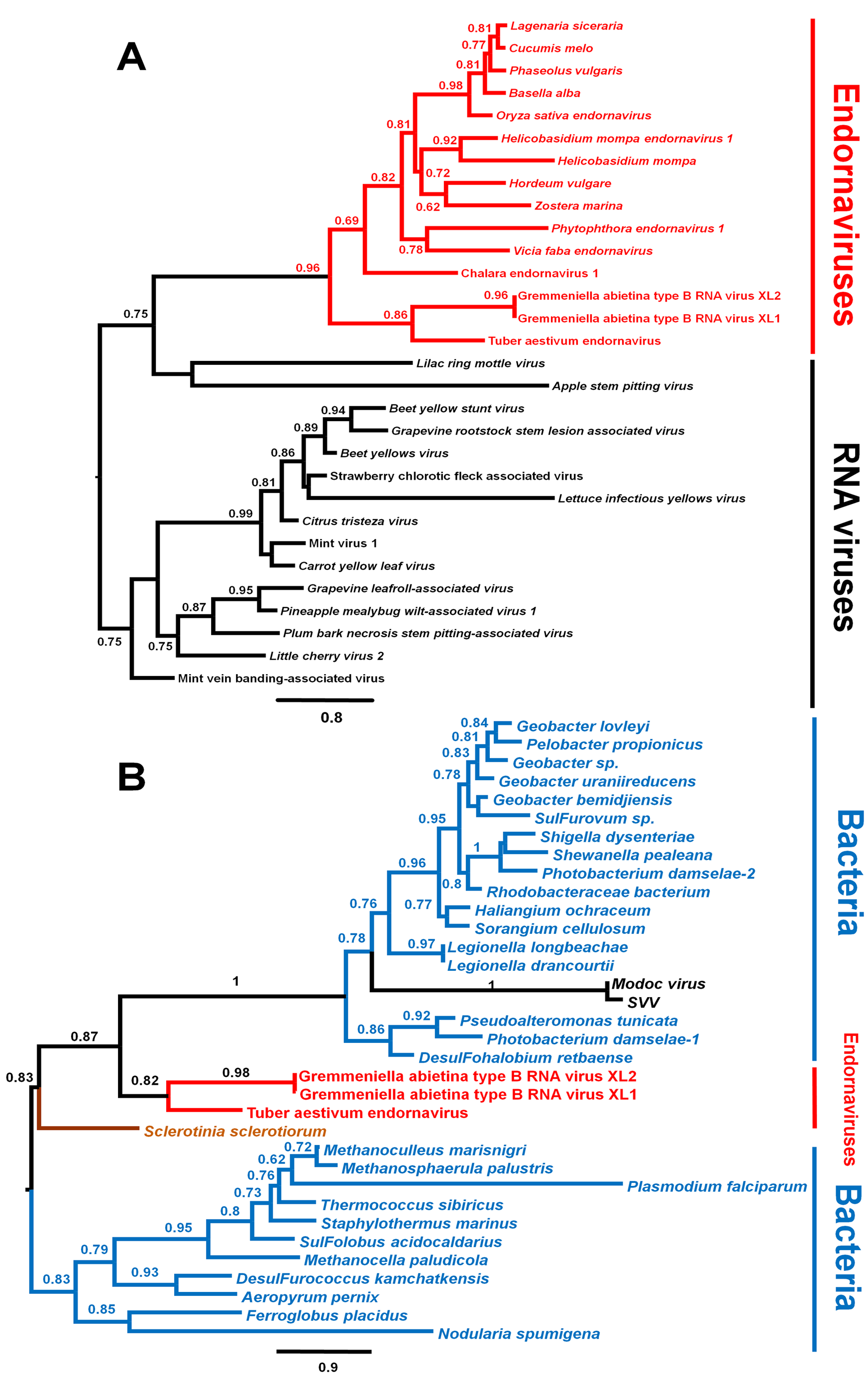

Supplement: Figure S3 — Phylogenetic relationships of endornaviruses and RNA viruses based on the RdRp and the helicase domain. (A) A phylogenetic tree was constructed based on the RdRp sequences of 15 endornaviruses and 15 RNA viruses. The sequences of only the RNA viruses whose RdRp sequences were highly similar to those of endornaviruses were selected. The red and black colors indicate endornaviruses and RNA viruses, respectively. (B) A phylogenetic tree was constructed based on DEAD-like helicase (DEXDc) domains derived from bacteria (in blue), endornaviruses (in red), a fungus (in brown), and other viruses (in black). Amino acid sequences highly homologous to the DEXDc sequences of three endornaviruses were used for the phylogenetic analysis. The aLRT values of each branch were calculated using a SH-like method, and values greater than 0.5 are shown. SVV is an abbreviation for Simian varicella virus. (TIF) [file pone.0064270.s003.tif]
